# Percutaneous Versus Surgical Interventions for Hepatic Cystic Echinococcosis: A Systematic Review and Meta-Analysis

**DOI:** 10.1007/s00270-021-02911-4

**Published:** 2021-07-16

**Authors:** G. L. E. Mönnink, C. Stijnis, O. M. van Delden, R. Spijker, M. P. Grobusch

**Affiliations:** 1 Department of Infectious Diseases, Division of Internal Medicine, Center of Tropical Medicine and Travel Medicine, Amsterdam Public Health, Amsterdam Infection & Immunity, Amsterdam University Medical Centers, Location AMC, University of Amsterdam, PO Box 22660, 1100 DD Amsterdam, The Netherlands; 2 Department of Radiology, Amsterdam University Medical Centers, Location AMC, University of Amsterdam, Amsterdam, The Netherlands; 3 Medical Library, Amsterdam Public Health, Amsterdam Infection & Immunity, Amsterdam University Medical Centers, location AMC, University of Amsterdam, Amsterdam, The Netherlands

**Keywords:** Cystic echinococcosis, Meta-analysis, Percutaneous procedures, Surgery, Systematic review

## Abstract

**Purpose:**

This systematic review and meta-analysis summarises the current literature on invasive treatment options of cystic hepatic echinococcosis (CE), comparing percutaneous radiological interventions to surgery, still the cornerstone of treatment in many countries.

**Methods:**

A literature search was conducted in Medline and EMBASE databases (PROSPERO registration number: CRD42019126150). The primary outcome was recurrence of cysts after treatment. Secondary outcomes were complications, duration of hospitalisation, mortality and treatment conversion.

**Results:**

The number of eligible prospective studies, in particular RCTs, was limited. In the four included studies, only conventional surgery is compared directly to percutaneous techniques. From the available data, in terms of recurrence, percutaneous treatment of hydatid cysts is non-inferior to open surgery. With regard to complications and length of hospital stay, outcomes favour percutaneous therapy.

**Conclusion:**

Although evidence from prospective research is small, percutaneous treatment in CE is an effective, safe and less invasive alternative to surgery.

**Supplementary Information:**

The online version contains supplementary material available at 10.1007/s00270-021-02911-4.

## Introduction

Echinococcosis, a group of zoonoses caused by cestodes of the genus *Echinococcus*, is one of currently twenty neglected tropical diseases (NTDs) [1]. With an estimated loss of over one million Disability Adjusted Life Years (DALY’s) annually worldwide, it imposes a high burden on health [2, 3]. Parasitologically, an infected human is a dead-end intermediate host, disrupting the life cycle. Clinically, human echinococcosis presents predominantly in three forms, each requiring a different therapeutic approach. The fox tapeworm *Echinococcus multilocularis* causes the alveolar type, also endemic to Western Europe [4]. The so-called neotropical echinococcoses, *E. vogeli and E.oligarthus*, are found exclusively in the rural areas of Central and South America, are hosted by bush dogs and may sporadically cause polycystic echinococcosis in humans [5–7].

This article will be focusing on the most prevalent type, cystic echinococcosis (CE), caused by the dog tapeworm *Echinococcus granulosus*.

CE is endemic to various regions worldwide [8]. In highly endemic regions, the prevalence of CE may exceed 5–10% of the population [9]. Prevention strategies include anthelmintic dog treatment, slaughter hygiene, surveillance, and health-educational measures [10]. However, it still remains a public health problem in certain regions, with an estimated 2–3 million cases worldwide and 19,300 deaths annually [3, 11]. Increasingly, imported cases from endemic areas are seen in Western Europe as a result of migration [12] Infection with *E. granulosus* may cause cysts in all organs, but does so predominantly in the liver (75%) [13, 14]*.* CE can remain clinically silent for several years. If left untreated, cysts may rupture and cause anaphylaxis, cysto-biliary fistulae and direct spread to the peritoneal cavity, or haematogenous spread to other organs, resulting in the development of new cysts [15]. The choice of treatment is often guided by the radiological stage of the disease, using the Gharbi or WHO classifications (see Table 1) [16]. Treatment may differ widely across different regions or countries, depending on local expertise and resources.

**Table 1:** Sonographic classification of hydatid cysts retrieved from Mohan et al. [16]

Gharbi type	WHO type	Cyst morphology
I	CE 1	Unilocular anechoic lesion with double line sign
III	CE 2	Multiseptated rosette-like honeycomb cyst
II	CE 3A	Cyst with detached membranes (water-lily sign)
III	CE 3B	Cyst with daughter cyst in solid matrix
IV	CE 4	Cyst with heterogeneous hypoechoic/hyperechoic contents. No daughter cysts
V	CE 5	Solid plus calcified wall

Several management options for CE are at hand: chemotherapy, surgery, percutaneous drainage and a ‘wait-and-watch’ approach. The latter approach is chosen in older, inactive (WHO CE4 or CE5) cysts [17, 18]*.* Backbone in most treatment strategies is chemotherapy with the anthelmintic drug albendazole. Chemotherapy is effective in early stages of uncomplicated CE and in reducing the risk of recurrence. Albendazole is administered several weeks to months [19, 20]. Adverse effects of long-term albendazole use are usually relatively mild; however, in some cases, severe hepatotoxicity occurs, prompting premature treatment cessation. In more advanced disease, unfortunately, as is the case in many patients at the time of diagnosis, albendazole alone is ineffective in curing or even preventing disease progression. Therefore, additional treatment options have been developed over the years. The surgical approach has long been the gold standard in treatment of CE. There are various surgical options: conservative, radical and laparoscopic [21]. A percutaneous treatment option is PAIR (puncture–aspiration–injection–reaspiration). This technique utilises ultrasound to guide puncture of the cyst, destruction of the germinal layer with protoscolices using a scolicidal agent, and reaspiration of this fluid after 15–20 min. PAIR is generally indicated in younger cysts, i.e. WHO types 1 and 3A or, respectively, Gharbi types 1 and 2 [22]. In our centre, a modified percutaneous technique, the so-called PEVAC (percutaneous evacuation of cyst content), is used in vital, more mature hydatid cysts (e.g. Gharbi type 3). Similar techniques have been developed in other treatment centres as an alternative to surgery [23].

To date, several studies on percutaneous treatment options and their outcomes have been published [23, 24].

The aim of this systematic review and meta-analysis is to directly compare percutaneous radiological interventions to surgery in CE. As abdominal CE is most common, and non-surgical interventions are almost exclusively performed in abdominal cysts, we limited our search to this clinical presentation.

## Methods

### Search Strategy and Selection Criteria

This systematic review with meta-analysis has been PROSPERO-registered (International Prospective Register of Systematic reviews with the following ID; CRD42019126150) and was reported according to PRISMA guidelines [25].

The first systematic search in the databases Medline and EMBASE was carried out on 1 May 2019 and repeated thirteen months later. An information specialist (RS) performed both database searches. No language- or study date restrictions were applied. Animal studies, case reports, reviews, conference abstracts, articles about extra-abdominal manifestations and articles about infection caused by other species than *Echinococcus granulosus* were excluded. Studies exclusively concerning children were excluded for methodological concerns. Retrospective cohort studies were excluded due to high selection bias risk. For a list of terms used in the search and details of the study selection, see Supplementary Figs. 1, 2a and 2b.

After the primary selection, articles were screened for title and abstract first and full text later. Two researchers (G.L.E.M. and C.S.) independently assessed the studies. Any conflicts regarding inclusion and exclusion of articles were resolved in consensus. The full selection process is depicted in a PRISMA flowchart (see Supplementary Figs. 2a and 2b). Data extraction was reported according to PRISMA guidelines. The primary outcome was recurrence of cysts after treatment. Secondary outcomes were complications, duration of hospitalisation, mortality and treatment conversion.

The following data were extracted: first author; year of publication; study type; intervention type; study population and number of patients; primary outcome; secondary outcome; the clinical implication and remarks on the studies included. For an overview of characteristics, see Supplementary Table 1. Both minor and major complications were included in our analysis.

The quality of the studies was assessed using the Cochrane RoB tool for RCTs, and the ROBINS-I tool for prospective cohort studies. The RoB tool identified five sources of bias: randomisation process, deviations from intended intervention, missing outcome data, measurement of the outcome and selection of the reported result. The ROBINS-I tool focused on: confounding, selection of participants, classification of intervention, deviations from intended intervention, missing data, measurement of outcomes and selection of the reported results as potential sources for bias. Critical appraisal was performed by two reviewers independently, and conflicts were resolved by discussion leading to consensus. Results of the quality assessment are summarised in Supplementary Figs. 3 and 4 [26].

### Data Analysis

From the four studies included in the systematic review, two RCTs were included in the meta-analysis. The other two, prospective cohort studies, were excluded from the meta-analysis because of high selection bias risk. RevManager was used to perform the meta-analysis. For the binary outcomes (recurrence and complications), fixed-effect models and 95% confidence intervals (CIs) were used. For continuous outcomes (i.e. length of hospital stay), fixed-effect models and weighted mean differences were used. An unsuccessful attempt was made to retrieve missing standard deviations for one study, Shera et al. [27], by contacting the corresponding author. Eventually, the standard deviations were estimated by using the ‘finding SD-calculator’ option of RevManager, assuming that standard deviations were equal in both intervention groups.

## Results

The online search provided 901 potentially relevant studies. After removal of duplicates, 700 studies remained potentially eligible. After screening for title and abstract, 685 articles were excluded. Supplementary Figs. 2a and 2b describe the full study selection process and reasons for exclusion. When necessary, external databases were used to retrieve full text. Two articles written in Russian were translated. Ultimately, a total of four studies with a total of 234 patients were included in the systematic review (Table 2). In two studies, the RoB assessment revealed a serious risk of bias; in the remaining two studies, both RCT, a moderate risk of bias. As no conversion of treatment or deaths were reported, these outcome measures are absent from our data analysis.

**Table 2:** Overview of results

	Khuroo et al.		Tan et al.		Abdelraouf et al.		Shera et al.	
Year of publication	1997		1998		2015		2017	
Number of patients	50		102		40		42	
Type of intervention	Percutaneous catheterisation	Surgery	PAIR	Surgery	PAIR	PAIR-S	DPAI	Surgery
Number of patients per intervention	25	25	36	66	23	17	21	21
Recurrence (*n*)	3	7	0	20	8	0	0	2
%	12%	28%	0	30.3%	34.8%	0%	0%	9%
Complication (number of patients)	8	21	4	26	3	1	2	8
%	32%	84%	11.1%	39.3%	13.0%	5.9%	9.5%	33.3%
Hospital stay (days)	4.2 $\pm$ 1.5	12.7 $\pm$ 6.5	5	18.5 $\pm$ 14.3	1–2	2–4	2.28	8.23
Mortality (*n*)	0	0	0	0	0	0	0	0
%	0%	0%	0%	0%	0%	0%	0%	0%

In a study conducted by Khuroo et al. in India [28] (1997), 25 patients were randomly assigned to percutaneous catheterisation using hypertonic saline (20%) and 25 patients to surgical treatment (cystectomy). Both groups consisted of 16 univesicular and 9 multivesicular cysts. Unfortunately, information on exact WHO stages was lacking. The primary outcome, recurrence, was lower in the percutaneous catheterisation group, 12% versus 28%, however, not statistically significant (OR = 2.9, 95% CI 0.6–10.6, *p* = 0.29). Secondary outcomes, complications (OR = 11.2, 95% CI 2.7–36.0, *p* < 0.001) and hospital stay (MD = 8.5, 95% CI 5.8–11.2, *p* < 0.001) were statistically significant, favouring the percutaneous group. In the surgery group, three major complications in two patients, i.e. two biliary rupture and one incisional hernia, resulted in surgical revision. One patient from the percutaneous group required surgery to treat the biliary rupture. In the early stages after percutaneous catheterisation, urticaria (*n* = 2) and transient hypotension (*n* = 1) were seen. More patients had a transient fever 24 h after surgery, i.e. *n* = 17 compared to *n* = 3 after percutaneous treatment (odds ratio 15.6; 95% CI 3.4–64.7; *P* < 0.001). Furthermore, this study showed that percutaneous drainage is effective in both univesicular and multivesicular cysts.

In 1998, Tan et al. [29] reported on a 5-year follow-up of 102 patients from the Surgery Department of Gülhane Military Medical Academy in Ankara, Turkey. The patients were divided into two groups. The first group consisted of 66 patients with Gharbi type III, IV and V cysts, treated with surgery. The second group consisted of 36 patients with Gharbi type I and II cysts, treated with PAIR, of which 15/36 were treated with PAIR with a single-episode catheterisation. After surgery, complications consisted of biliary fistula (*n* = 10), wound infection (*n* = 7), pleural effusion (*n* = 6), abscess formation in the cystic space (*n* = 2) and cholangitis (*n* = 1). Complications in the PAIR group were only seen when PAIR with catheterisation was performed. In four patients, biliary fistulae developed.

Percutaneous treatment combined with medical treatment in this study proved a successful alternative to surgery for Gharbi type 1, type 2 and some selected type 3 hydatid cysts of the liver, also resulting in fewer complications, lower recurrence rate and shorter hospitalisation period.

In a cohort of 54 patients from Egypt, Abdelraouf et al. [30] (2015) described three treatment arms, of which the first, conventional surgery, was retrospective. The patients in this group were considered historical controls and therefore not included in our statistical analysis. Both other arms, PAIR and PAIR followed by surgical de-roofing (called PAIR-S in the following) of the cyst, were prospectively followed up for seven years. In a total of 40 patients divided over the latter groups, PAIR-S showed no recurrence, whereas PAIR alone showed a recurrence rate of 34%, which was also higher than the historical controls. The complication rate was lowest in the PAIR-S group (5,9%; level of statistical significance not indicated), whereas the PAIR-alone group (13%) performed better than the historical surgical controls (42,9%). In both groups, the only complications mentioned were wound infections.

A study conducted in India by Shera et al. [27] (2017) compared surgery to DPAI (double percutaneous aspiration injection), an aspiration technique that is repeated after 3–7 days, with the intention to eliminate scolices which may have survived the first treatment weeks. Both arms contained 21 randomly assigned patients each. All cysts were staged as WHO 1 (16) and 3a (5) in the surgical arm, and 18 and 3 in the DPAI arm, respectively.

There was no single recurrence in the DPAI arm, versus 9.5% in the surgical arm. Complications were fewer, and duration of hospital stay was shorter than after surgery. High-grade fever/sepsis (*n* = 2), bile leak (*n* = 2) and sub-phrenic collection (*n* = 1) were seen as complications after surgery. After DPAI, one patient developed a pneumothorax that was treated conservatively. Altogether, the authors conclude that DPAI is non-inferior to surgery in the treatment of CE with advantages of shorter hospital stay, minimal invasiveness, shorter convalescence period and lower adverse effects.

**Fig. 1:**
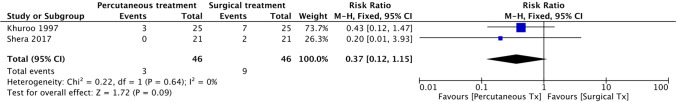
Forest plot on the relative risk of recurrence after percutaneous evacuation and surgery

**Fig. 2:**
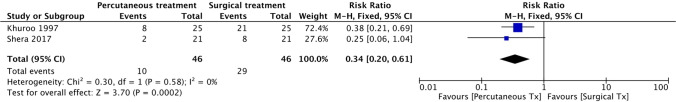
Forest plot on the relative risk of complications after percutaneous evacuation and surgery

**Fig. 3:**
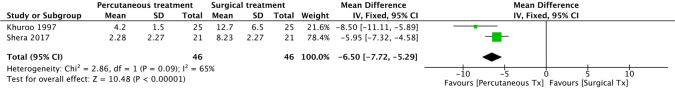
Forest plot on the mean difference in duration of hospital stay after percutaneous evacuation and surgery

In addition, we performed a meta-analysis with two RCTs (*n* = 92) on recurrence, post-operative complication rate and duration of hospital stay (Figs. 1, 2 and 3, respectively) [27, 28]. In the recurrence analysis, the pooled estimates suggested a lower, but statistically non-significant, recurrence rate after percutaneous procedure (RR 0.37, 95% CI 0.12 to 1.15). Additionally, pooled estimates on the risk of complications after intervention showed favourable results in the percutaneous procedure group. The relative risk of complications is statistically significantly lower after percutaneous procedure compared to surgery (RR 0.34, 95% CI 0.20–0.61). Lastly, the results of the meta-analysis revealed a shorter duration of mean hospital stay of 6.5 days after percutaneous procedure compared to surgery (weighted MD − 6.5, 95% CI − 7.72 to − 5.29 d.)

## Discussion

We compared surgery to percutaneous techniques in the treatment of vital hydatid cysts. As stated by the WHO Informal Working Group on Echinococcosis and by Brunetti et al. in their review, there is no best treatment option for CE [31, 32]. Decision on treatment of CE is based on stage-specific approach, hinging on imaging (i.e. ultrasound) characteristics. Many studies, most of which single centre, describe retrospective results of their treatment strategies. However, we were interested in the direct comparison between percutaneous treatment and surgery. Therefore, only prospective studies comparing these treatment options were eligible.

The studies included in this systematic review almost all show results favouring percutaneous radiological interventions as treatment for CE. In the direct comparison of percutaneous treatment compared to surgical cyst removal, recurrence rate was lower in most studies; fewer complications occurred; and a shorter hospital stay was observed. Only Abdelraouf et al. showed a higher recurrence rate after PAIR compared to PAIR combined with surgery (PAIR-S) [30].

These findings are in line with previously published data. A systematic review and meta-analysis conducted by Sokouthi et al. [33], comparing percutaneous treatment options to laparoscopy in CE, included 56 studies in their meta-analysis, of which fifty retrospective- and six prospective cohort studies. This study yielded favourable results for PAIR, with a lower post-operative complication rate, lower mortality rate and a higher cure rate. However, recurrence rates were lower for the laparoscopic group. No data on length of hospital stay was reported. Another systematic review conducted in 2003 by Smego et al. [34] compared the outcomes of 769 CE patients, treated with PAIR + albendazole or mebendazole, to 952 time period-matched (1990–2001) historical control subjects who received surgical intervention. Smego et al. also reported less recurrence (*P* < 0.0001; no confidence intervals provided), fewer major and minor complications (*P* < 0.0001), a lower mortality rate (*P* < 0.0824) and a shorter hospital stay (*P* < 0.001) in the PAIR + albendazole or mebendazole group. However, the results of the retrospective studies summarised in both systematic reviews may be biased by selection in favour of PAIR, as they compared patients treated with PAIR to a retrospective surgical control group.

To date, there is no consensus regarding the risk of recurrence after the various treatment options. According to the literature, less invasive surgical techniques coincide with higher recurrence rate and thus potential additional interventions [35]. After PAIR, Sokouthi et al. reported higher recurrence rates, whereas Smego et al. showed favourable recurrence rates in the PAIR + albendazole or mebendazole group [33, 34]. Also, in this systematic review, ambiguous results on recurrence rates were seen in the various studies. In the study with the longest follow-up (Abdelraouf et al.), recurrence rates were higher in the PAIR group [30]. However, our meta-analysis showed favourable results regarding recurrence rates in the percutaneous treatment group, although these results were not statistically significant. Apart from the length of follow-up, this discrepancy may be explained by increased experience when percutaneous treatment is frequently performed in a single centre.

The length of follow-up is considered to importantly influence the recurrence rate [36]. As shown in Table 3, there is a large bandwidth, ranging from 9 to 84 months, in the duration of follow-up in the included studies. This potentially biased the reported recurrence rates. Abdelraouf et al., with the longest period of follow-up, showed a higher recurrence rate after the PAIR procedure [30].

**Table 3:** Overview of period of follow-up per trial

	Khuroo et al.		Tan et al.		Abdelraouf et al.		Shera et al.	
Intervention	Percutaneous catheterisation	Surgery	PAIR	Surgery	PAIR	PAIR-S	DPAI	Surgery
Mean follow-up (months)	17.5 ± 7.0	17.4 ± 6.5	36	36	84	84	26	27.71
Range	9–24	9–24	–	–	–	–	18–36	18–36

One of the major limitations of this systematic review is the small number of included studies, because of the exclusion of all retrospective studies to prevent selection bias. However, a certain degree of selection bias was also identified in both included prospective cohort studies, as treatment options were not randomly assigned to the patients. This resulted in relatively difficult and/or multivesicular cysts being treated with surgery, consequently favouring the results of percutaneous treatment.

The size of all included studies is small, with a total of 234 patients analysed in four studies. Also, the studies yielded a clinical diversity, i.e. variability in included patients, both percutaneous and surgical interventions, and outcomes studied. There was great variety in what the different studies reported as minor and major complications. The forest plot on the relative risk of complications (Fig. 2) is a consequence of our choice to include all types of complications and might overestimate the actual relative risk of severe complications in surgery.

Apart from this, there is methodological heterogeneity. Some studies administered albendazole prior to both interventions, whereas other studies limited this to the percutaneous treatment group only. Another study performed the percutaneous treatment twice with an interval of 3–7 days to minimise the risk of recurrence.

To the best of our knowledge, this is the first systematic review and meta-analysis directly comparing surgery to percutaneous treatment of echinococcal cysts in prospective studies. Although numbers are small, results suggest favourable outcomes of percutaneous treatment in the absence of selection bias, inevitable in retrospective and to a lesser extent in non-RCT prospective studies.

This study emphasises the importance of comparing different treatment options after randomization in equally staged cysts, preferably in the framework of a multicentre setting and a sufficient period of follow-up.

## Conclusion

To date, consensus about the treatment of CE is lacking. Contrary to popular belief, less invasive treatment methods, using percutaneous evacuation to treat CE, appear to be at least non-inferior in terms of recurrence rate and significantly show less post-operative complications and shorter hospital stay compared to surgical treatment.

## Supplementary Information

Below is the link to the electronic supplementary material.

Supplementary file1 (DOCX 1057 KB)
