# Supplementary material for: Percutaneous Versus Surgical Interventions for Hepatic Cystic Echinococcosis: A Systematic Review and Meta-Analysis
Source: Cardiovasc Intervent Radiol. 2021 Jul 16;44(11):1689–96. doi: 10.1007/s00270-021-02911-4 (PMC8550455; doi:10.1007/s00270-021-02911-4)
Supplement: Supplementary file 1 — Supplementary file1 (DOCX 1057 KB) [file 270_2021_2911_MOESM1_ESM.docx]

| Title  **Table S1** Overview of studies included in systematic review. | Authors | Year | Study type | Study population | Intervention | Number of patients | Recurrence  (n) | Results secondary outcome (n) | Clinical implication | Remarks |
| --- | --- | --- | --- | --- | --- | --- | --- | --- | --- | --- |
| Clinical and serological outcomes with different surgical approaches for human hepatic hydatidosis | Abdelraouf *et al.* | 2015 | Cohort study**,**  Mixed retrospective and prospective. | Patients with isolated hepatic hydatid cysts aged 22-54 years. | Group 1 = 14 patients underwent radical surgical removal.  Group 2 = 23 patients underwent PAIR.  Group 3 = 17 patients underwent PAIR followed by deroofing and cyst evacuation  (PAIR-S).  (combined surgical procedure)  All patients were administered albendazole tablets 400 mg twice daily for two weeks before and six months after the procedure. | n=54 | Group 1:  n=2  Group 2:  n=8  Group 3:  n=0 | Group 1:  Complications: n=6  2 post-operative bleeding and 4 post-operative wound infection  Mortality: 3 (anaphylactic shock)  Hospital days: 10-21 days  Conversions: not mentioned  Group 2:  Complications: n=3  3 post-operative wound infection  Mortality: n=0  Hospital days: 1-2 days  Conversions: not mentioned  Group 3:  Complications: n=1  1 post-operative wound infection  Mortality: n=0  Hospital days: 2-4 days  Conversions: not mentioned | According to the authors, the partial surgical procedure; PAIR followed by deroofing and evacuation is the least invasive method with lowest rates of complications and mortality. | This study is partly retrospective, comparing the retrospective surgical cohort with a prospective non-surgical cohort.  The PAIR-S technique is used in large (>5cm cysts) or a failed PAIR procedure.  Furthermore, the concentration of the hypertonic solution used in the PAIR-S procedure is 16-20%. It’s unclear what the concentration of the hypertonic solution used in the PAIR procedure is. |
| Percutaneous drainage compared with surgery for hepatic hydatid cysts | Khuroo *et al.* | 1997 | Prospective randomized controlled trial | Patients with hepatic CE randomly assigned to percutaneous catheterisation or cystectomy ≥14 years of age. | Percutaneous catheterisation = 25 patients  Cystectomy = 25 patients  Albendazole (10 mg per kg for 8 weeks) was administered to patients who underwent the percutaneous catheterisation procedure | n=50 | PAIR:  n=3  Cystectomy: n=7  (P=0.29) | Percutaneous catheterisation:  Complications: n=8  1 transient hypotension, 2 urticaria  3 fever <24 hours after procedure, 1 cyst infection (late complication)**,** 1 biliary rupture  Mortality: n=0  Hospital days: 4.2±1.5 days P<0.001  Conversions: n=0  Cystectomy:  Complications: n=21 (P<0.001)  17 fever <24 hours after procedure, 1 cyst infection (early complication), 1 prolonged tube drainage, 2 biliary fistula, 1 incisional hernia  Mortality: n=0  Hospital days: 12.7±6.5 days  Conversions: n=0 | This study shows that percutaneous drainage is effective for both univesicular and multivesicular cysts. The efficacy of percutaneous drainage is similar to that of standard cystectomy. The advantages of percutaneous catheterisation include a shorter hospital stay and a lower complication rate | No albendazole treatment was given to patients treated with cystectomy. |
| A comparison of imaging guided double percutaneous aspiration injection and surgery in the treatment of cystic echinococcosis of liver | Shera *et al* | 2017 | Prospective randomised controlled trial | Patients older than 12 years with isolated hepatic hydatid cysts  (no statistically significant difference between the two groups) | DPAI= 21 patients were treated with double percutaneous aspiration injection. Patients in this group were put on dexamethasone 2 mg bid for 2 days  Surgery (n= 21)  Cystectomy with tube drainage (n=16)  Cystectomy with capitonnage (n=3)  Cystectomy with omentopexy (n=2)  All patients were put on albendazole (10 mg/kg/day) for 1 week before either procedure | N=42 | DPAI n=0  Surgery n=2 | DPAI:  Complications: n=2  1 high-grade fever/sepsis, 1 pneumothorax.  Mortality: n=0  Hospital days: 2.28 days  Conversions: n=0  Surgery:  Complications: n=8  2 sepsis/high-grade fever, 2 bile leak, 1 subphrenic collection, 1 prolonged tube drainage, 1 low-grade fever, 1 wound infection.  Mortality: n=0  Hospital days: 8.23 (5-14 days) P<0.001  Conversions: n=0 | This study shows that DPAI is non-inferior to surgery with advantages of shorter hospital stay, minimal invasiveness, shorter convalescence period and lower adverse effects.  The advantage of DPAI is that the procedure is repeated after 3-7 days which helps to eliminate scolices which may have survived the first dose. | All complications in the surgery group were seen to occur in patients undergoing cystectomy with tube drainage.  All patients with multivesicular cysts were excluded.  Response was successful in 95.3% (n=20) in the DPAI group. In the surgery group, response was successful in 85.7% (n=18).  At the initial puncture of one patient from the DPAI-arm, the aspirated fluid was positive for bilirubin on the dipstick, procedure was abandoned, and the patient was excluded from the study.  In 18 patients, the procedure was performed under USG guidance and in rest of the patients (n=3), CT guidance was used. |
| The results of surgical treatment and percutaneous drainage of hepatic hydatid disease | Tan *et al.* | 1998 | Prospective cohort study | Patients diagnosed with hydatid disease of the liver between 1992-1997. | Surgery (66 patients with 79 cysts);  Partial cystectomy+ capitonage + omentoplasty  (n=20)  Partial cystectomy + external drainage  (n=49)  Pericystectomy (n=8)  Cystojejunostomy (n=2)  PAIR (n=36):  PAIR (n=21)  PAIR + cath.  (n=15)  Albendazole (10 mg/kg/day) was started 7 days prior to PAIR and continued 3 weeks after PAIR. | N=102 | Surgery:  n=20  PAIR:  n=0  (P<0.05) | Surgery:  Complications: n=26  10 biliary fistula formation, 7 wound infection, 6 pleural effusion, 2 abscess formation in the cystic space, and 1 incident of cholangitis.  Mortality: n=0  Hospital days: 18.5 days ± 14.3 (range 6-60 days)  Conversions: not mentioned  Percutaneous drainage:  Complications: n=4  4 biliary fistula formations.  Mortality: n=0  Hospital days: 5 days (p<0.05)  Conversions: not mentioned | According to Tan *et al.* the larger the diameters of the cysts the greater the number of complications found (P<0.05). However, no statistical difference was found between the cyst’s diameter and recurrence rate (P>0.05)  Percutaneous drainage + medical treatment is a successful alternative to surgery for type 1, type 2 and some selected type 3 hydatid cysts of the liver. Resulting in less complications, lower recurrence rate and shorter hospitalization period. | Percutaneous drainage was performed on cysts <5 cm and surgical treatment was carried out for cysts >5cm. Furthermore, generally, solitary cysts (type I and II) were treated by percutaneous drainage and multiple cysts (type II, IV, V) were treated by surgery. |

**Table S2**. PRISMA Checklist.

| **Section/topic** | **#** | **Checklist item** | **Reported on page #** |
| --- | --- | --- | --- |
| **TITLE** | | |  |
| Title | 1 | Identify the report as a systematic review, meta-analysis, or both. |  |
| **ABSTRACT** | | |  |
| Structured summary | 2 | Provide a structured summary including, as applicable: background; objectives; data sources; study eligibility criteria, participants, and interventions; study appraisal and synthesis methods; results; limitations; conclusions and implications of key findings; systematic review registration number. |  |
| **INTRODUCTION** | | |  |
| Rationale | 3 | Describe the rationale for the review in the context of what is already known. |  |
| Objectives | 4 | Provide an explicit statement of questions being addressed with reference to participants, interventions, comparisons, outcomes, and study design (PICOS). |  |
| **METHODS** | | |  |
| Protocol and registration | 5 | Indicate if a review protocol exists, if and where it can be accessed (e.g., Web address), and, if available, provide registration information including registration number. |  |
| Eligibility criteria | 6 | Specify study characteristics (e.g., PICOS, length of follow-up) and report characteristics (e.g., years considered, language, publication status) used as criteria for eligibility, giving rationale. |  |
| Information sources | 7 | Describe all information sources (e.g., databases with dates of coverage, contact with study authors to identify additional studies) in the search and date last searched. |  |
| Search | 8 | Present full electronic search strategy for at least one database, including any limits used, such that it could be repeated. |  |
| Study selection | 9 | State the process for selecting studies (i.e., screening, eligibility, included in systematic review, and, if applicable, included in the meta-analysis). |  |
| Data collection process | 10 | Describe method of data extraction from reports (e.g., piloted forms, independently, in duplicate) and any processes for obtaining and confirming data from investigators. |  |
| Data items | 11 | List and define all variables for which data were sought (e.g., PICOS, funding sources) and any assumptions and simplifications made. |  |
| Risk of bias in individual studies | 12 | Describe methods used for assessing risk of bias of individual studies (including specification of whether this was done at the study or outcome level), and how this information is to be used in any data synthesis. |  |
| Summary measures | 13 | State the principal summary measures (e.g., risk ratio, difference in means). |  |
| Synthesis of results | 14 | Describe the methods of handling data and combining results of studies, if done, including measures of consistency (e.g., I^2^) for each meta-analysis. |  |

| **Section/topic** | **#** | **Checklist item** | **Reported on page #** |
| --- | --- | --- | --- |
| Risk of bias across studies | 15 | Specify any assessment of risk of bias that may affect the cumulative evidence (e.g., publication bias, selective reporting within studies). |  |
| Additional analyses | 16 | Describe methods of additional analyses (e.g., sensitivity or subgroup analyses, meta-regression), if done, indicating which were pre-specified. |  |
| **RESULTS** | | |  |
| Study selection | 17 | Give numbers of studies screened, assessed for eligibility, and included in the review, with reasons for exclusions at each stage, ideally with a flow diagram. |  |
| Study characteristics | 18 | For each study, present characteristics for which data were extracted (e.g., study size, PICOS, follow-up period) and provide the citations. |  |
| Risk of bias within studies | 19 | Present data on risk of bias of each study and, if available, any outcome level assessment (see item 12). |  |
| Results of individual studies | 20 | For all outcomes considered (benefits or harms), present, for each study: (a) simple summary data for each intervention group (b) effect estimates and confidence intervals, ideally with a forest plot. |  |
| Synthesis of results | 21 | Present results of each meta-analysis done, including confidence intervals and measures of consistency. |  |
| Risk of bias across studies | 22 | Present results of any assessment of risk of bias across studies (see Item 15). |  |
| Additional analysis | 23 | Give results of additional analyses, if done (e.g., sensitivity or subgroup analyses, meta-regression (see Item 16) |  |
| **DISCUSSION** | | |  |
| Summary of evidence | 24 | Summarize the main findings including the strength of evidence for each main outcome; consider their relevance to key groups (e.g., healthcare providers, users, and policy makers). |  |
| Limitations | 25 | Discuss limitations at study and outcome level (e.g., risk of bias), and at review-level (e.g., incomplete retrieval of identified research, reporting bias). |  |
| Conclusions | 26 | Provide a general interpretation of the results in the context of other evidence, and implications for future research. |  |
| **FUNDING** | | |  |
| Funding | 27 | Describe sources of funding for the systematic review and other support (e.g., supply of data); role of funders for the systematic review. |  |


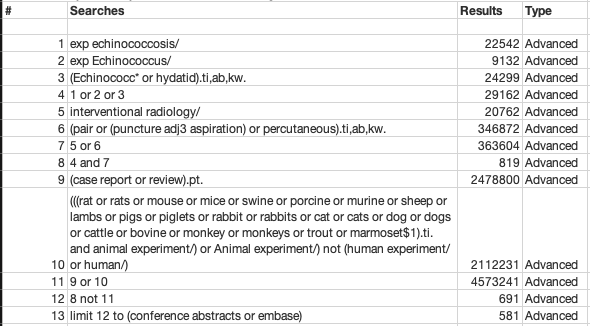


**Figure S1.** Full search strategy.

**Figure S2.** Paper selection flow chart first search.


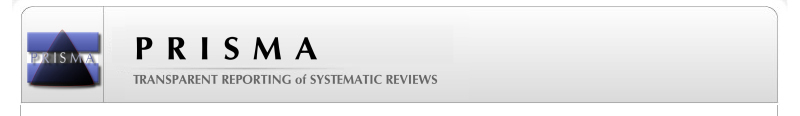
**PRISMA 2009 Flow Diagram**

Full-text articles excluded, with reasons (n = 11)

- N=2; articles focused exclusively on paediatric patients
- N=2; only (congress) abstract
- N=1; article focused on anthelmintic drug treatment solely.
- N=1; article focused on
- N=1; article was a prospective epidemiology study with no relevant endpoints.
- N=4; articles turned out to be retrospective.

Studies included in quantitative synthesis (meta-analysis)
(n = 2)

Studies included in qualitative synthesis
(n = 4)

Full-text articles assessed for eligibility
(n =15)

Records excluded
(n = 685)

Records screened
(n = 700)

Records after duplicates removed
(n = 700)

Additional records identified through other sources.
(n = 0 )

Records identified through first database searching
(n = 901)

Identification

Screening

Eligibility

Included

**Figure S3.** Paper selection flow chart second search.


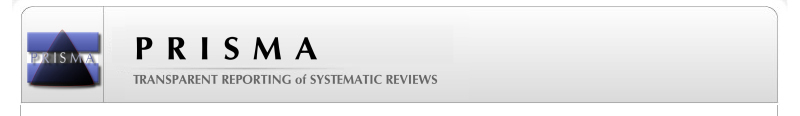
**PRISMA 2009 Flow Diagram**

Full-text articles excluded, with reasons (n = 2)

- N=2; retrospective cohort studies.

Records after duplicates with first search removed
(n = 48)

Studies included in quantitative synthesis (meta-analysis)
(n = 0)

Studies included in qualitative synthesis
(n = 0)

Full-text articles assessed for eligibility
(n =2)

Records excluded
(n = 46)

Records screened
(n = 48)

Additional records identified through other sources
(n = 0)

Records identified through database searching
(n = 954)

Identification

Screening

Eligibility

Included

**Figure S4**. Risk of bias assessment non – randomized studies using Risk of Bias Visualisation [26]


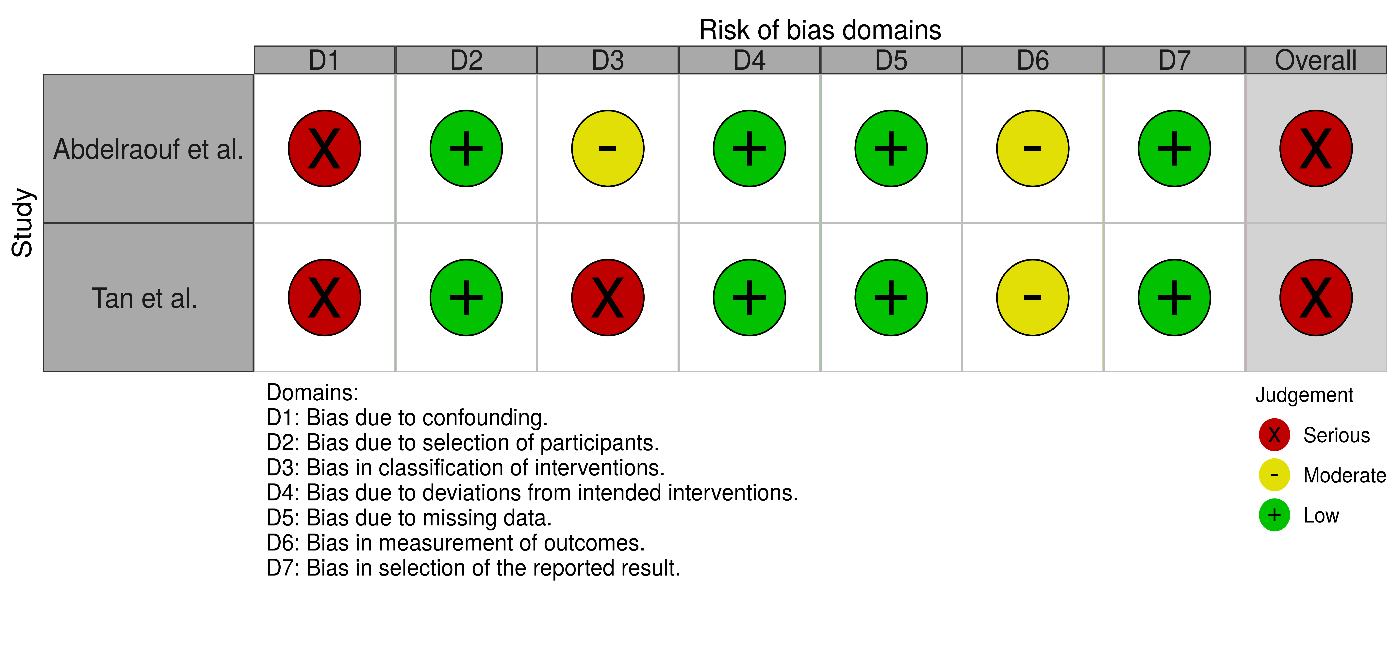


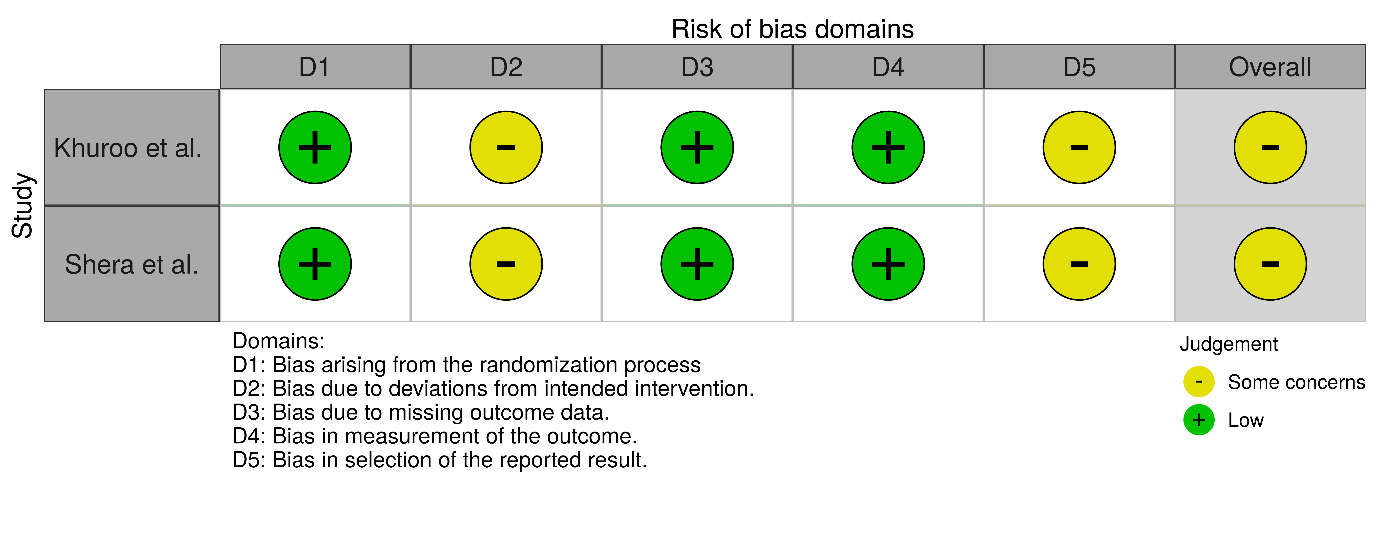
**Figure S5.** Risk of bias assessment of RCT studies using Risk Of Bias Visualisation [26]
